# FRESH: Long-Term Outcomes of a Randomized Trial to Reduce Radon and Tobacco Smoke in the Home

**DOI:** 10.5888/pcd16.180634

**Published:** 2019-09-12

**Authors:** Ellen J. Hahn, Amanda T. Wiggins, Kathy Rademacher, Karen M. Butler, Luz Huntington-Moskos, Mary Kay Rayens

**Affiliations:** 1 University of Kentucky Colleges of Nursing and Public Health, Lexington, Kentucky; 2 University of Kentucky College of Nursing, Lexington, Kentucky

## Abstract

**Introduction:**

Tobacco smoke and radon are the leading causes of lung cancer. The FRESH intervention was a randomized controlled trial of 515 homeowners to promote stage of action to reduce radon and air nicotine levels.

**Methods:**

We studied 515 participants, 257 in a treatment group and 258 in a control group. Treatment participants received free radon and air nicotine test kits, report back, and telephone support, and those participants whose homes had high radon levels received a voucher for $600 toward mitigation. Both groups were asked to retest 15 months post intervention. We examined differences in stage of action to test for and mitigate radon and adopt a smoke-free–home policy and in observed radon and air nicotine values by study group over time.

**Results:**

Homeowners in the treatment group scored higher on stage of action to test for radon and air nicotine and to mitigate for radon during follow-up than those in the control group at 3 months and 9 months, but the effect of the intervention diminished after 9 months. We saw no difference between groups or over time in observed radon or air nicotine values. Of homeowners in the treatment group with high radon levels at baseline, 17% mitigated, and 80% of them used the voucher we provided.

**Conclusion:**

The null finding of no significant change in observed radon or air nicotine values from baseline to 15 months may reflect the low proportion of radon mitigation systems installed and the decline in stage of action to adopt a smoke-free home policy. Including a booster session at 9 months post intervention may improve the remediation rate.

**Summary**

**What is already known on this topic?**

Tobacco smoke and radon are major causes of lung cancer. However, few US residents view radon as an immediate health risk, and few test their homes for radon.

**What is added by this report?**

We provide results of a randomized controlled trial testing the efficacy of providing free in-person home radon and air nicotine test kits coupled with report back and a telephone problem-solving session as a means of reducing lung cancer risk.

**What are the implications for public health practice?**

The treatment effect was maintained for 9 months post intervention, suggesting a window of opportunity to promote radon mitigation or adoption of a smoke-free home policy.

## Introduction

Approximately 221,200 new cases of lung cancer occur annually in the United States (1). Tobacco smoke and radon exposure are the 2 leading causes of lung cancer (2), and exposure to both (ie, synergistic risk) heightens the probability of developing the disease (3). The lifetime risk of radon-induced lung cancer is 62 per 1,000 ever-smokers versus 7 per 1,000 never-smokers (4). Exposure to radon may be more harmful for never-smokers exposed to secondhand smoke (5).

Residential radon exposure is a significant, modifiable risk factor for lung cancer death worldwide (6). However, few US residents view radon as an immediate health risk (7), despite high radon levels in 1 in 15 residences (8). Because radon is a colorless, odorless gas, many fail to recognize the potential for home exposure. In 1 study, 82% of respondents had heard of radon but only 15% had tested for its presence (9). In other research, rural family medical offices distributed 746 radon detection kits, but only 55% were returned (10). Because exposure to tobacco smoke plus radon increases lung cancer risk nearly tenfold (8), interventions are needed to reduce these risks.

Optimal reduction of risk from exposure to radon and secondhand smoke requires testing for radon and mitigating exposure if radon levels are elevated and adopting a smoke-free home policy. A radon mitigation system installed by a certified radon professional can reduce radon exposure (8). Our objective was to test the effects of an intervention consisting of home-testing for radon and secondhand smoke and personalized report back to the participant by trained research staff members. We assessed stages of action (11) for radon testing and radon mitigation and for air nicotine testing and adopting a smoke-free home. We hypothesized that homeowners who received their radon and air nicotine results and telephone-based problem solving would score higher on stages of action to test and remediate for radon and secondhand smoke, controlling for personal characteristics, compared with those who did not receive the intervention. We also hypothesized that observed radon and air nicotine values of participants in the treatment group would be lower post intervention than at baseline.

## Methods

We assessed stage of action for radon testing and radon mitigation and for air nicotine testing and adopting a smoke-free home policy via a self-report survey at 3 months, 9 months, and 15 months post intervention. The study period, from first enrollment to last data collection, was January 2013 through August 2017.

### Design and sample

Our target sample size was 275 participants per group before data collection (95% power to detect a medium effect size in the main effects of group and time and their interaction with an α level of .05). Recruitment ended just short of this goal (N = 515), but the robust degree of power for these planned comparisons suggested adequate enrollment. We divided participants between a treatment group (n = 257) and a control group (n = 258) by using stratified quota sampling. In each group, half of participants had 1 or more smokers in the home and half had no smokers in the home. Trained research staff members screened for eligibility, enrolled participants, randomly assigned them to a study group on site, administered the baseline survey, and delivered the first phase of the intervention in person. 

Study participants were adults aged 21 or older with access to a telephone who could speak and read English and owned a single-family home that had not been tested for radon in the past 2 years. Only 1 participant per household was eligible for the study. Participants were recruited from central Kentucky primary care clinics, a pharmacy, and at community events. Age, sex, race/ethnicity, education, and employment status did not differ by recruitment location. We invited participants to complete subsequent surveys even if they had missed a previous one. The study was approved by the University of Kentucky institutional review board.

### Intervention

FRESH (Freedom from Radon Exposure and Smoking in the Home) was a 2-step intervention. In the first step, we provided free radon and air nicotine test kits to the treatment group for home testing along with verbal, written, and YouTube video instructions for using the kits. Second, we consulted with participants by telephone to report back the test results and to help them solve problems related to high radon or air nicotine levels. To measure radon, we used short-term test kits from Air Chek, Inc (http://www.radon.com/). Participants sent the kit to the Air Chek laboratory in a postage-paid envelope. We assessed secondhand smoke exposure by using passive airborne nicotine samplers (12), which we sent to the Johns Hopkins School of Public Health Environmental Health and Engineering laboratory for analysis. Approximately 11 weeks after participants completed testing, trained research staff members conducted 20-to-25–minute telephone problem-solving sessions by using a standardized report-back protocol to assess the Precaution Adoption Process Model (PAPM) stage and the participant’s response to the test results. Researchers delivered queries (ie, asked questions) and messages tailored to the PAPM stage to share strategies for lowering radon (ie, mitigation) and secondhand smoke exposure (ie, adopting a smoke-free home policy). All participants in the treatment group whose homes tested high for radon received a voucher for $600 toward the cost of radon mitigation. The cost of radon mitigation depends on how the home is built and the extent of the radon problem (8). Participants in the control group could request free test kits (simulating standard public health practice) from the research team at a later date following enrollment in the study.

### Measures

We categorized participants’ stage of action to test and remediate homes for radon and secondhand smoke as 1) unaware, 2) unengaged, 3) deciding, 4) action, and 5) maintenance. For ease of interpretation, we combined the original PAPM stages 3 to 5 (3, deciding about acting; 4, decided not to act; 5, decided to act) to define deciding. Researchers often combine PAPM stages depending on specific health behaviors (13). 

**Stage of action.** We evaluated stage of action at baseline and at 3-month, 9-month, and 15-month intervals by using multiple survey questions for both radon and secondhand smoke and asked separate questions for testing and remediation. The scoring algorithm for the 4 stages of action measures are described elsewhere (14). At baseline, participants were not in maintenance for radon or air nicotine testing (a study requirement was not having been tested for radon in the past 2 years, and air nicotine tests were not commercially available). Scoring at each follow-up assessment was based on responses to the same stage of action questions and whether they had tested since baseline.

**Radon and air nicotine values.** Participants in the treatment group were given free short-term radon and air nicotine test kits and asked to test their homes at baseline. Participants in the control group could request test kits after enrollment. At 15 months post intervention, all study participants were mailed free test kits for both air contaminants. Baseline radon and air nicotine values were used to assign participants to risk groups (ie, tested high, tested low, or did not test/invalid result). We also used these test data to evaluate changes in radon and air nicotine levels from baseline to 15 months. Given skewness in the distributions, these values were log-transformed before analysis.

**Teachable moment constructs.** Lung cancer worry was assessed by using a 4-question scale (15). The first question (“How much do you currently worry about getting lung cancer someday?”) was rated from 1 (not at all) to 5 (all of the time). The remaining 3 questions, including “How much do worries about lung cancer impact your mood?” were rated on a 4-point scale, from 1 (not at all) to 4 (a lot). Each of the questions was standardized by subtracting the overall mean from the individual score and dividing this difference by the overall standard deviation; these were then summed to represent overall lung cancer worry, with higher scores signifying greater worry (16). Lung cancer risk was measured by asking: “How would you rate your risk of developing lung cancer in your lifetime on a scale of 0 to 10?” Higher scores indicated greater perceived risk (17). Synergistic risk was measured by using a question that rated the perceived risk from being exposed to radon and smoking a pack of cigarettes per day compared with the risk of smoking a pack of cigarettes a day with no radon exposure on a 5-point scale ranging from 1 (much less risky) to 5 (much more risky). Health-related self-concept was measured by using the 8-question health-protective motivation subscale of the Health-Related Self Concept scale (18) to assess beliefs and attitudes toward health-enhancing behaviors and behavioral intentions. Responses were on a 7-point scale ranging from 1 (disagree entirely) to 7 (agree entirely). The negatively worded item (eg, “Generally, I am careless of my health”) was reverse-coded before summing the items; higher scores indicated greater health-related self-concept. Cronbach’s α was 0.91.

**Self-efficacy.** We measured self-efficacy by using a 3-question scale that measured ability (“I am able to test my home for radon to prevent lung cancer”), resources (“I have the time to test”), and ease of action (“I can easily test”) (19) to assess confidence in taking each of 4 health actions: testing for radon, mitigating radon, testing for air nicotine, and adopting a smoke-free home policy. Respondents rated the 3 questions on a 5-point scale ranging from 1 (strongly disagree) to 5 (strongly agree). Self-efficacy scores were determined for each action. Cronbach’s α were ≥0.83 for all 4 actions.

**Smoking in the home**. We assessed smoking in the home by asking, “Do you or any other members of your household smoke cigarettes, cigars, or pipes?” We collected demographic and personal factors on all study participants (age, sex, race/ethnicity, education, employment status, years living in current residence, and family history of lung cancer).

**Risk status for radon and air nicotine.** We categorized baseline test results as “tested high,” “tested low,” or ”did not test/invalid result.” On the basis of the Environmental Protection Agency action level for radon (8), home values at or above 4.0 picocuries per liter (pCi/L) were considered to test high for radon. Air nicotine values greater than 0.1 μg/m^3^ were considered to test high for secondhand smoke (20).

### Data analysis

Baseline comparisons between study groups and between completers and dropouts (ie, noncompleters) were made by using the 2-sample *t* tests or χ^2^ tests of association. Linear mixed modeling evaluated the variables associated with changes over time in stages of action for testing and remediation for radon and secondhand smoke. Similarly, we assessed the factors associated with differences in radon and air nicotine log-transformed testing values at baseline and at 15 months. Baseline demographic and teachable-moment factors were included as covariates. Lung cancer worry and risk, synergistic risk, health-related self-concept, and self-efficacy were measured at each assessment (3 months, 9 months, 15 months) and included as time-dependent covariates in each model. Remediation models were also adjusted for risk status. The smoke-free home adoption model included only those participants with smokers in the home. The 4 stages of action models each had a significant interaction between treatment and time, rendering the main effects not interpretable; post-hoc pairwise comparisons were done on the interaction effects by using Fisher’s least significant difference procedure. Data analysis was conducted using SAS, version 9.4 (SAS Institute Inc) with α = .05.

## Results

**Figure 1:**
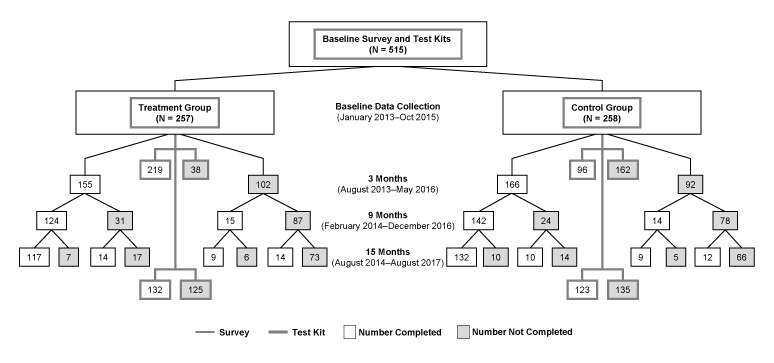
Flow diagram of enrollment and completion of surveys and testing for the baseline, 3-month, 9-month, and 15-month assessments of the FRESH (Freedom from Radon Exposure and Smoking in the Home) randomized controlled trial to reduce radon and secondhand smoke exposure in the home, Central Kentucky, Jan 2013–August 2017.

The mean age of participants was 51 years. Most were non-Hispanic white women with college degrees (Table 1). Nearly one-fourth had a family history of lung cancer. Consistent with stratification, half lived with at least 1 smoker. Most (85.2%) participants in the treatment group completed baseline radon and air nicotine testing (Figure 1). Fewer (37.2%) in the control group completed testing. We maintained approximately 60% retention throughout the study (Figure 1). There was no difference in retention between treatment and controls at any follow-up (*P* > .10 for each χ^2^ test comparison). 

**Table 1:** Baseline Characteristics of Participants (N = 515) in the FRESH Trial and Comparison of Completers and Noncompleters, Central Kentucky, January 2013–August 2017^a^

Characteristic	Potential Range^b^	Total sample (N = 515)^c^	Completers (n = 317)^c^	Noncompleters (n = 198)^c^	*P* Value
**Age, mean (SD)**	—	51.2 (12.7)	52.4 (12.4)	49.4 (12.9)	.009^d^
**Sex**					
Male	—	166 (32.2)	101 (31.9)	65 (32.8)	.82^e^
Female	—	349 (67.8)	216 (68.1)	133 (67.2)	
**Race/ethnicity**					
Non-Hispanic white	—	437 (85.2)	273 (86.7)	164 (82.8)	.23^e^
Non-white or Hispanic	—	76 (14.8)	42 (13.3)	34 (17.2)	
**Education**					
Less than college degree	—	199 (38.7)	99 (31.3)	100 (50.5)	<.001^e^
College degree	—	315 (61.3)	217 (68.7)	98 (49.5)	
**Employed for wages**					
Yes	—	308 (59.9)	197 (62.1)	111 (56.3)	.19^e^
No	—	206 (40.1)	120 (37.9)	86 (43.7)	
**Years living in home, mean (SD)**	—	13.3 (10.9)	14.1 (10.8)	12.3 (11.0)	.07^d^
**Family history of lung cancer**					
Yes	—	123 (23.9)	67 (21.1)	56 (28.3)	.06^e^
No	—	392 (76.1)	250 (78.9)	142 (71.7)	
**Smokers in the home**					
Yes	—	256 (49.7)	141 (44.5)	115 (58.1)	.003^e^
No	—	259 (50.3)	176 (55.5)	83 (41.9)	
**Study group**					
Treatment	—	257 (49.9)	154 (48.6)	103 (52.0)	.45^e^
Control	—	258 (50.1)	163 (51.4)	95 (48.0)	
**Self-efficacy, radon testing, mean (SD)**	5–15	13.1 (2.2)	13.2 (2.1)	12.9 (2.3)	.11^d^
**Radon mitigation, mean (SD)**	5–15	10.4 (2.7)	10.6 (2.7)	10.0 (2.7)	.02^d^
**Secondhand smoke testing, mean (SD)**	5–15	12.9 (2.2)	13.0 (2.1)	12.7 (2.2)	.13^d^
**Adopting a smoke-free policy, mean (SD)**	5–15	13.8 (2.7)	14.0 (2.6)	13.4 (2.8)	.02^d^
**Lung cancer worry,^f^ mean (SD)**	−3 to 14^c^	<0.1 (3.2)	−0.6 (2.7)	1.0 (3.7)	<.001^d^
**Lung cancer risk, mean (SD)**	0–10	3.8 (2.5)	3.5 (2.4)	4.4 (2.6)	<.001^d^
**Synergistic risk, mean (SD)**	1–5	3.8 (1.0)	4.0 (0.9)	3.7 (1.0)	.002^d^
**Health related self-concept, mean (SD)**	8–56	46.4 (8.2)	47.5 (7.6)	44.7 (9.0)	<.001^d^
**Risk group, radon^g^**					
High	—	92 (17.9)	73 (23.0)	19 (9.6)	<.001^e^
Low	—	178 (34.6)	138 (43.5)	40 (20.2)	
Invalid or did not test	—	245 (47.5)	106 (33.4)	139 (70.2)	
**Risk group, secondhand smoke**					
High	—	66 (12.8)	43 (13.6)	23 (11.6)	<.001^e^
Low	—	184 (35.7)	146 (46.1)	38 (19.2)	
Invalid or did not test	—	265 (51.5)	128 (40.4)	137 (69.2)	

Abbreviations: —, not applicable; SD, standard deviation.

a A randomized controlled trial of 515 homeowners to promote stage of action to reduce radon and air nicotine levels. Completers and noncompleters refer to participants surveyed at 15 months to assess stage of action for radon testing and radon mitigation and for air nicotine testing and adopting a smoke-free home.

b Range of scores depending on the self-report scale.

c Values are number (percentage) unless otherwise indicated. Percentages may not sum to 100 because of rounding. For some variables, the number of observations does not total to the column total because of missing data for a small number of participants.

d Calculated by using 2-sample *t* test.

e Calculated by using χ^2^ test of association.

f Because the number of response options for the lung cancer worry questions was not uniform across items, each question was standardized by subtracting the mean and dividing by the standard deviation prior to adding the items together. For this reason, the range for lung cancer worry includes negative values and the mean is close to 0.

g There were 3 risk groups, based on baseline testing, for each of radon and air nicotine: those who tested high, those who tested low, or those who did not test or who had an invalid result.

**Baseline study group differences.** We saw few differences in the study variables between the treatment and control groups at baseline with the exception of self-efficacy for radon testing (*P* = .004, with treatment exceeding controls by an average of 0.6) and risk group for each of radon and secondhand smoke. Risk group differences were significant because treatment group participants were more likely than controls to test for baseline radon and air nicotine (*P* < .001 for both). Among those who tested, we saw no difference between treatment and control groups in the proportion of test results that were high for either contaminant. We saw no differences between the treatment and control groups at baseline for either employment status or the length of time in current residence.

**Differences between completers and noncompleters.** Participants who completed the 15-month survey were older than noncompleters and more likely to have a college degree and not report smokers living in the home. Completers also had lower scores for lung cancer worry and risk but higher scores for synergistic risk perception and health-related self-concept (Table 1). Completers were more likely to have tested for the contaminants at baseline (59.7%) than noncompleters (30.8%; χ^2^ = 40.5, *P* < .001).

**Figure 2:**
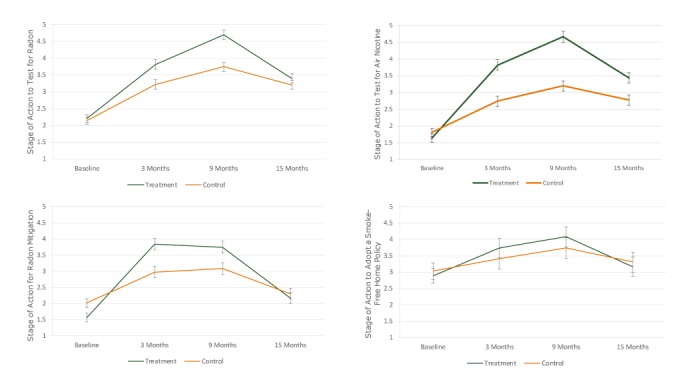
Adjusted means and 95% confidence intervals for the treatment and control groups on stage of action for testing and remediation outcomes for the baseline, 3-month, 9-month, and 15-month assessments of the FRESH (Freedom from Radon Exposure and Smoking in the Home) randomized controlled trial to reduce radon and secondhand smoke exposure in the home, Central Kentucky, January 2013–Aug 2017. Group means from models were adjusted for age, sex, race/ethnicity, education, employment, time living in current residence, smoking in the home, self-efficacy, lung cancer worry, lung cancer risk, synergistic risk, and health related self-concept. Brackets indicate confidence intervals.

**Predictors of radon testing stage of action.** Participants who were non-Hispanic white and had greater self-efficacy had higher stage-of-action scores for radon testing than nonwhite and Hispanic participants and those with lower self-efficacy (Table 2) (Figure 2). Post-hoc analysis indicated the groups did not differ at baseline (*P* = .460) or 15 months (*P* = .052), but the treatment group had a higher average compared with controls at both the 3-month and 9-month assessments (*P* < .001 for both comparisons) (Figure 2). Within each group, there was an increase in this outcome between baseline and 3 months and between 3 months and 9 months, followed by a decrease between 9 and 15 months (*P* < .001 for each comparison).

**Table 2:** Differences in Stages of Action for Radon Testing and Mitigation, Participants in Treatment (n = 257) and Control (n = 258) Groups by Participant Characteristics, Calculated by Linear Mixed Models, the FRESH Intervention,^a^ Central Kentucky, January 2013–August 2017

Variable	Stage of Action^b^			
	Radon Testing (n = 499)		Radon Mitigation (n = 499)	
	F	*P* Value^c^	F	*P* Value^c^
Age	2.73	.098	1.43	.23
Male	0.35	.55	<0.01	.96
Non-Hispanic white	4.00	.046	0.07	.80
College education	1.64	.20	1.09	.30
Employed for wages	1.20	.27	1.78	.18
Years living in home	0.50	.48	2.05	.15
Family history of lung cancer	0.32	.57	0.03	.86
Smokers in the home	2.89	.089	1.13	.29
Self-efficacy	47.29	<.001	36.18	<.001
Lung cancer worry	0.04	.83	0.23	.63
Lung cancer risk	<0.01	.97	0.88	.35
Synergistic risk	3.21	.074	15.42	<.001
Health-related self-concept	3.82	.051	0.13	.71
Risk group	—	—	85.73	<.001
Time	426.88^b^	<.001	226.62^b^	<.001
Treatment	49.90^b^	<.001	9.57^b^	.002
Treatment × time	23.21	<.001	34.12	<.001

Abbreviation: —, not applicable.

a FRESH was a randomized controlled trial to promote stage of action to reduce radon and air nicotine levels in the home.

b Regressions modeling stage of action to test for radon and radon mitigation ranging from 1 (unaware) to 5 (maintenance over time). Main effects were not interpretable in either model given the presence of a significant interaction effect; means for the interaction effect are shown in Figure 2. Although the full sample size was 515, 16 participants missing 1 or more variables in the models could not be included in the multivariable analysis.

c *P* values calculated by type 3 tests of fixed effects in the mixed models.

**Table:** 

Stage of Action	Treatment, Mean (95% CI)	Control, Mean (95% CI)	*P* Value
Radon testing			
Baseline	2.20 (2.09–2.32)	2.14 (2.03–2.26)	.46
3 months	3.81 (3.67–3.95)	3.22 (3.07–3.36)	<.001
9 months	4.79 (4.55–4.84)	3.74 (3.60–3.88)	<.001
15 months	3.40 (3.26–3.53)	3.20 (3.07–3.34)	.052
Radon mitigation			
Baseline	1.57 (1.43–1.70)	2.01 (1.87–2.16)	<.001
3 months	3.83 (3.65–4.01)	2.98 (2.80–3.15)	<.001
9 months	3.75 (3.56–3.94)	3.09 (2.91–3.27)	<.001
15 months	2.17 (1.96–2.35)	2.30 (2.13–2.47)	.31
Air nicotine testing			
Baseline	1.64 (1.52–1.76)	1.80 (1.68–1.92)	.054
3 months	3.82 (3.66–3.98)	2.74 (2.58–2.90)	<.001
9 months	4.66 (4.49–4.83)	3.20 (3.04–3.35)	<.001
15 months	3.44 (3.29–3.59)	2.77 (2.62–2.92)	<.001
Adopting a smoke-free home policy			
Baseline	2.88 (2.65–3.12)	3.03 (2.78–3.29)	.41
3 months	3.74 (3.44–4.03)	3.41 (3.09–3.73)	.15
9 months	4.08 (3.77–4.39)	3.74 (3.43–4.06)	.14
15 months	3.17 (2.87–3.46)	3.31 (3.00–3.61)	.52

**Predictors of radon mitigation stage of action.** Participants who had greater self-efficacy to mitigate radon exposure and who perceived greater synergistic risk were at a higher stage of action for radon mitigation relative to those with lower self-efficacy and with lower perceived synergistic risk scores (Table 2) (Figure 2). Compared with those who tested low for radon at baseline, those who tested high or did not test at all were at a lower stage of action to mitigate (*P* < .001 for both). Stage of action to mitigate was lower for treatment than controls at baseline (*P* < .001), but the group averages at 3 and 9 months were significantly higher for treatment than control (*P* < .001 for both) (Figure 2). By 15 months, there was no group difference (*P* = .31). Within each group, the increase from baseline to 3 months and the decrease from 9 to 15 months were significant (*P* < .001 for each comparison), but the change from 3 to 9 months was not (*P* ≥ .34 for each group).

**Predictors of air nicotine testing stage of action.** Participants with smokers in the home and those with greater self-efficacy reported a higher average stage of action to test for air nicotine (Table 3) (Figure 2). Based on post-hoc testing, the 2 study groups were similar in stage of action for air nicotine testing at baseline (*P* = .054), but treatment exceeded controls at each follow-up (*P* < .001 for each comparison) (Figure 2). Within each group, we observed a significant increase from baseline to 3 months and another increase from 3 to 9 months, followed by a decrease from 9 to 15 months (*P* < .001 for each comparison).

**Table 3:** Differences in Stages of Action for Air Nicotine Testing and Adopting a Smoke-Free Home Policy, Participants in Treatment (n = 257) and Control (n = 258) Groups by Participant Characteristics, Calculated by Linear Mixed Models, the FRESH Intervention,^a^ Central Kentucky, January 2013–August 2017

Characteristic	Stage of Action^b^			
	Secondhand Smoke Testing (n = 499)		Adopting a Smoke-Free Home Policy (n^c^ = 247)	
	F	*P* Value^d^	F	*P* Value^d^
Age	1.53	.22	0.02	.89
Male	0.46	.50	0.09	.77
White/non-Hispanic	1.18	.28	0.15	.70
College education	0.64	.42	3.61	.058
Employed for wages	0.29	.59	0.36	.55
Years living in home	0.02	.88	0.24	.63
Family history of lung cancer	0.12	.72	0.81	.37
Smokers in the home	26.41	<.001	—	—
Self-efficacy	52.72	<.001	31.79	<.001
Lung cancer worry	1.06	.30	0.01	.92
Lung cancer risk	<0.01	.96	1.65	.20
Synergistic risk	2.00	.16	2.20	.14
Health-related self-concept	<0.01	.99	0.41	.52
Risk group	—	—	5.01	.007
Time	439.97^e^	<.001	29.33^e^	<.001
Treatment	126.78^e^	<.001	0.32^e^	.57
Treatment x time	63.01	<.001	3.14	.026

Abbreviation: —, not applicable; FRESH, Freedom from Radon Exposure and Smoking in the Home intervention.

a FRESH was a randomized controlled trial to promote stage of action to reduce radon and air nicotine levels in the home.

b Although the full sample size was 515, 16 participants missing 1 or more variables in the models could not be included in the multivariable analysis.

c This model was restricted to those with smokers in the home (n = 256; 9 were omitted because of missing 1 or more variable values in the model).

d *P* values calculated by type 3 tests of fixed effects in the mixed models.

e Regressions modeling stage of action to test for secondhand smoke and adopt a smoke-free home ranging from 1 (unaware) to 5 (maintenance over time). Main effects not interpretable in either model given the presence of a significant interaction effect.

**Predictors of smoke-free home policy adoption stage of action.** Among participants living with at least one smoker, those with at least a college degree and greater self-efficacy were more ready to adopt a smoke-free home; however, those with high baseline air nicotine were less ready to do so. Treatment and control groups did not differ on this outcome at any time point (*P* > .14 for each) (Figure 2). Within each group, there was an increase from baseline to 3 months (*P* ≤ .013 for both) and a decrease from 9 to 15 months (*P* ≤ .01 for both). From 3 to 9 months, there was an increase in stage of action to adopt a smoke-free home policy among treatment participants (*P* = .04), but the change from 3 to 9 months among controls was not significant (*P* = .054). 

**Predictors of radon and air nicotine.** Observed home radon values ranged from 0.3 to 35.0 at baseline and 0.3 to 23.8 at 15 months. Observed air nicotine values ranged from 0.003 to 21.8 at baseline and from 0.005 to 21.4 at 15 months. The radon and air nicotine models contained the same covariates and fixed effects as the stage of action models for testing. Though each model was significant overall, the main and interaction effects for group (treatment vs control) and time (baseline vs 15 months) were not significant in either. Higher average radon level was associated with lower lung cancer worry, whereas higher average air nicotine level was associated with having smokers in the home, higher lung cancer worry, and lower health-related self-concept.

Of the 59 homeowners in the treatment group with high radon levels at baseline, 10 (17%) self-reported mitigating for radon at the end of the study; 8 of these redeemed vouchers. Of the 33 control group participants with high radon levels at baseline, 6 (18%) self-reported mitigating at study completion. Among those with smokers in the home, 58% of treatment and 55% of control participants reported adopting a smoke-free home policy at the end of the study. Neither of these remediation outcomes demonstrated a significant study group effect.

## Discussion

Homeowners who received the FRESH intervention scored higher on stages of action to test for radon and secondhand smoke and to mitigate for radon at 3-month and 9-month follow-ups than those who did not, but by 15 months post intervention the group differences in these outcomes were no longer significant. Treatment group participants had higher stages of action to test for air nicotine even at 15 months, but we saw no differences in stage of action to adopt a smoke-free home policy between the groups at any time point. The decrease in stages of action between 9 and 15 months, regardless of outcome, suggests that the intervention had a diminishing effect by study end.

We planned the 15-month follow-up to provide ample time to mitigate. However, only 17% of participants in the treatment group with high radon levels reported mitigating their homes, even with a voucher to defray the cost. Control group participants reported the same mitigation rate (with no vouchers provided). We saw no significant change in radon or air nicotine values from baseline to 15 months. This null finding may reflect the low proportion of radon mitigation systems installed and the decline in stage of action to adopt and maintain a smoke-free home policy over time. Although stage of action to test and mitigate for radon improved over time, those most at risk (those with high radon levels) had low remediation rates, and the intervention did not affect actual remediation. Including a booster with an emphasis on radon mitigation for people with high radon levels after 9 months may improve the remediation rate, thereby decreasing exposure. Further research is needed to examine the disconnect between readiness to take action and actual remediation to reduce environmental exposure.

Treatment participants acquired the free test kits in person at baseline as an intervention component. Controls were asked to call for a free test kit at a later date (simulating standard public health practice). Home testing among treatment participants at baseline far exceeded what would be expected using standard practice (21). This highlights the value of distributing test kits in ambulatory health care settings to boost the likelihood of dual (radon and air nicotine) home screening. Comparative effectiveness research is needed to evaluate testing rates when test kits are distributed in alternative sites such as libraries or schools. One study determined that social marketing messages using digital signage technology in health departments was effective in increasing radon program participation (22).

In addition to the effects of the FRESH intervention, self-efficacy was a significant predictor for each stage-of-action outcome. Those who believed that they had the ability to test or remediate were more likely to indicate readiness to take action. This underscores the need for providing clear instructions and interventions to boost confidence in lung cancer prevention activities, such as using YouTube videos, easy-to-understand test kit instructions, and strategies to reduce barriers (including cost) to mitigating for radon and adopting a smoke-free home. Given that the monetary vouchers had a modest effect on the rate of radon mitigation, promoting access to low-interest loan providers and discount pricing provided by mitigation companies could increase affordable radon mitigation, which may enhance self-efficacy for this outcome.

Participants with high radon and air nicotine levels at baseline had lower stage-of-action scores for radon mitigation and for adopting a smoke-free home over time. Further research is needed to understand the complex psychological factors and other barriers to remediation among people whose homes test positive for environmental exposures (23). Interestingly, increased synergistic risk perception was a significant predictor of higher stage-of-action scores for radon mitigation over time. This has implications for public health education and community awareness, because the general public is not alert to the health risks of radon exposure or the combined risk of exposure to radon and tobacco smoke (7,9). Perceived risk creates a teachable moment that can lead to behavior change (24). In the case of lung cancer prevention, our findings support the critical role of informing the public of the risk of tobacco smoke plus radon in prompting action to reduce exposure. Health care providers need to speak with patients about these combined environmental hazards (25). Further investigation is needed to understand the role of perceived synergistic risk, if any, in prompting adoption of smoke-free home policies. As public health and health care systems integrate lung cancer risk reduction messaging, synergistic risk perception and its association with action to adopt smoke-free home policies must be evaluated.

Participants who did not complete the study scored higher on baseline lung cancer worry and perceived risk, and lower for synergistic risk and health-related self-concept. Previous studies noted that cancer worry and perceived risk are differentially associated with the avoidance of health behaviors related to screening and health maintenance (26). Further research is needed to explore the barriers to radon testing and mitigation among people with lung cancer worry and high perceived risk, such as the challenges posed by identifying a certified radon professional, scheduling and completing the mitigation process, and arranging mitigation payment. The addition of a booster session after 9 months could allow an opportunity to tailor the intervention to people who experience greater lung cancer worry or perceived risk.

Our study had strengths and limitations. A strength of the study design was its large sample and its stratification by home smoking. The intervention, easily delivered in primary care settings, was shown to be an effective way to promote home testing and remediation. A study limitation was attrition, as with most longitudinal trials, although we had consistent retention throughout the follow-up regardless of study group. Another limitation was that our sample was predominantly non-Hispanic white people with a college degree, so these results may not be broadly generalizable. Future studies would benefit from a more diverse sample. Furthermore, we did not evaluate cigarette pack-years among current and former smokers. Smoking history may affect study outcomes. This limitation is somewhat reduced because home smoking status was not associated with stage of action to test or mitigate for radon. Finally, because radon and air nicotine measurements may be higher in cold months (27), the report-back intervention may be more effective if initial radon and air nicotine testing is timed for when these contaminants are likely to be highest.

Providing free test kits in person in primary care settings, telephone support, and report back of results improved readiness to take action to remediate for radon and secondhand smoke. This low-cost intervention would likely benefit from a booster session at 9 months and a mechanism for linking people with high home radon levels with financial assistance for mitigation. These enhancements to our intervention would increase self-efficacy to take action to remediate the home for radon and secondhand smoke.
